# A Spatial Simulation Model for the Dispersal of the Bluetongue Vector *Culicoides brevitarsis* in Australia

**DOI:** 10.1371/journal.pone.0104646

**Published:** 2014-08-08

**Authors:** Joel K. Kelso, George J. Milne

**Affiliations:** School of Computer Science and Software Engineering, University of Western Australia, Crawley, Western Australia, Australia; Technische Universität Dresden, Medical Faculty, Germany

## Abstract

**Background:**

The spread of Bluetongue virus (BTV) among ruminants is caused by movement of infected host animals or by movement of infected *Culicoides* midges, the vector of BTV. Biologically plausible models of *Culicoides* dispersal are necessary for predicting the spread of BTV and are important for planning control and eradication strategies.

**Methods:**

A spatially-explicit simulation model which captures the two underlying population mechanisms, population dynamics and movement, was developed using extensive data from a trapping program for *C. brevitarsis* on the east coast of Australia. A realistic midge flight sub-model was developed and the annual incursion and population establishment of *C. brevitarsis* was simulated. Data from the literature was used to parameterise the model.

**Results:**

The model was shown to reproduce the spread of *C. brevitarsis* southwards along the east Australian coastline in spring, from an endemic population to the north. Such incursions were shown to be reliant on wind-dispersal; *Culicoides* midge active flight on its own was not capable of achieving known rates of southern spread, nor was re-emergence of southern populations due to overwintering larvae. Data from midge trapping programmes were used to qualitatively validate the resulting simulation model.

**Conclusions:**

The model described in this paper is intended to form the vector component of an extended model that will also include BTV transmission. A model of midge movement and population dynamics has been developed in sufficient detail such that the extended model may be used to evaluate the timing and extent of BTV outbreaks. This extended model could then be used as a platform for addressing the effectiveness of spatially targeted vaccination strategies or animal movement bans as BTV spread mitigation measures, or the impact of climate change on the risk and extent of outbreaks. These questions involving incursive *Culicoides* spread cannot be simply addressed with non-spatial models.

## Introduction

The past decade has seen the development of increasingly detailed simulation models aimed at capturing the transmission dynamics of directly transmitted diseases, such as Foot and Mouth Disease and Classical Swine Fever in livestock [1]–[4] and human pandemic influenza [5]–[7]. Such models have been used to establish the effectiveness of intervention strategies and to develop containment and control strategies (e.g. for human pandemic influenza [5]–[7]) and eradication strategies (e.g. for Foot and Mouth Disease [1]).

The development and use of mathematical disease models of insect-vectored human diseases dates back over a century to the work by Ross on malaria transmission [8]. However, the development of data-rich simulation models for insect-vectored diseases has advanced more slowly, mainly due to the additional complexity inherent in representing the dynamics of both host and vector populations, and pathogen transmission between them. An additional layer of complexity is introduced if the goal is to model the spatial spread of a pathogen over a landscape since both vector movement and habitat-dependent insect vector abundance potentially affect spatial disease spread. Faster moving vectors clearly have the potential to increase the rate of disease spread; but disease spread may also depend on the population density of vectors, since greater vector numbers mean greater transmission of pathogen between vectors and host as well as greater numbers of vectors moving to new locations. For example, high densities of mosquitos in particular locations are known to lead to disease transmission ‘hot spots’ and are often the focus of targeted control measures for mosquito vectored diseases [9]. Hence spatial vector population features need to be realistically modelled within a modelling environment if it is to be used to analyse the effectiveness of spatially targeted intervention strategies.

In this paper we describe and apply a model that couples insect vector dispersal with climate dependent insect vector population dynamics, with the goal of modelling vector-born disease spread in areas that exhibit what we refer to as an *incursive* vector population. By an incursive vector population, we mean that the presence or absence of vectors in different parts of the landscape can change seasonally or from year-to-year in a way that depends on vector introduction and dispersal.

Our motivating example of an incursive vector population is the biting midge *Culicoides brevitarsis* which is present in northern and eastern Australia and is a vector for several viral livestock diseases, including Bluetongue, which is caused by Bluetongue Virus (BTV), and Akabane [10]. *C. brevitarsis* survival and activity is temperature dependent; *C. brevitarsis* is present throughout the year in northern areas of New South Wales (NSW), but is unable to survive the cold winter period in southern areas [11], [12]. The spatial distribution of *C. brevitarsis* within NSW thus varies seasonally during the year, and from year to year, depending on spatiotemporal variation in temperature and wind, as midges are transported from northern areas into more southerly areas where they establish breeding populations in warmer months, and become locally extinct over winter. This *Culicoides* movement scenario is also reflective of past, well-documented incursions of *C. imicola* carrying BTV into the Balearic Islands (Spain) from North Africa [13], the probable wind dispersal of *Culicoides* between Greece, Turkey and Bulgaria [14] and what may occur if a new (to Australia) competent vector were to arrive from Indonesia, East Timor or Papua New Guinea and establish itself in Australia, see [15].

Incursive vector populations may be contrasted with *endemic* populations. An endemic population is permanently established and a breeding cycle will be sustained without introduction of transported population, even if the population falls to very low levels. The *Culicoides obsoletus* group BTV vectors are an endemic population in Northern Europe, which carried outbreaks of BTV in the summers of 2008 and 2009. In these outbreaks, once the warming created a vector population capable of sustaining BTV transmission, BTV spread was limited only by vector movement and not by temperature-dependent vector population dynamics.

The distinction between incursive and endemic vector populations is very significant from a modelling perspective, as the endemic scenario allows the simplifying assumption that vector population distribution does not depend upon vector movement, and so can be treated as a static model input. In the incursive vector scenario, this assumption is invalid, and both vector population dynamics and dispersal need to be modelled in tandem.

A simulation modelling methodology which permits spatially-explicit modelling of wind and flight movement of *C. brevitarsis* dispersal, together with its habitat and climate dependent population dynamics, is presented. The particular incursive scenario in coastal NSW is used to illustrate the development and application of the modelling methodology. Extensive trapping over the past four decades has resulted in high quality data which reports the arrival time of *C. brevitarsis* as it spreads southwards along the NSW coastal plain [11], [16]–[20]. These data permit the simulated midge incursions produced by the model to be validated by comparison with the field-derived datasets, and these results are presented. For other Culicoides species their specific habitat- and temperature-dependent characteristics would need to be modelled and the simulation model presented here re-parameterised. This is a region without a resident population of competent BTV midge vectors. *C. brevitarsis* is a competent tropical midge species common to northern Australia but populations of this midge cannot be sustained in most of NSW due to low winter temperatures. Annual incursions occur from endemic areas to the north, as the temperature increases in spring. This southward incursion scenario is significant in that it may act as a BTV conduit, allowing the virus to spread from endemic regions to the north to vulnerable, disease-free sheep-rearing areas in south-east Australia.

### 1.1 Background

Various species of *Culicoides* biting midges are present throughout the world with the exception of Antarctica and New Zealand. Where they are present, *Culicoides* can act as vectors of BTV, Akabane virus (and other viruses of the Orthobunyviridea family) and African Horse Sickness [10]. Competent *Culicoides* species are those capable of viral transmission, with susceptible female midges becoming infected following blood-feeding by biting an infectious ruminant host animal. As trans-ovarial BTV transmission (where infected females transfer the virus to their offspring) is unknown in *Culicoides*, onward animal infection occurs when infectious midges subsequently feed a second time on susceptible, uninfected animals.

Bluetongue is a significant disease from an animal health and economic perspective world-wide. In cattle, BTV infection is generally asymptomatic but its presence limits export to certain markets. In sheep, BTV infection is symptomatic and induces significant mortality rates, with 30% mortality rates recorded for epidemics in Spain, for example [21]. BTV is endemic in northern Australia but disease free status exists in the southern sheep rearing regions. The vectors considered to be most important in Australia are *C. fulvus*, which is an efficient vector but is restricted to areas with high summer rainfall and does not occur in the drier sheep-rearing areas of Australia; *C. wadai*, which is also an efficient vector that probably spread from Indonesia in the 1970s and has extended its range from an initial area near Darwin into Western Australia, Queensland, and New South Wales; and *C. brevitarsis*, which is a competent but inefficient vector for the BTV strains present in Australia but is more abundant than *C. fulvus* or *C. wadai* [10]. *C. brevitarsis* is a midge species that relies on cattle dung for ovipositioning (i.e. egg laying) and is endemic in northern Australia, but not in the sheep-rearing areas of Southern Australia. In an Australian setting, the future spread of BTV may be impacted by changes to weather patterns [22], the incursion of new competent insect vectors from Asia (i.e. new species of *Culicoides* midge carried by the wind) [15] or the incursion of new serotypes of BTV (which might have different competence characteristics) from Asia via midge dispersal. The risk to both commercial export markets and animal health caused by high mortality rates in sheep populations makes Bluetongue a disease of significance.

The spread and establishment of BTV in northern Europe [23] demonstrated a previously unforeseen ability of BTV to become endemic in cooler climates. This has resulted in increased research activities in virus transmission, on detailed reporting of these outbreaks [24], [25] and in initiation of modelling studies, for example [26]–[28].

*Culidoides* midges are known to be dispersed by the wind, often over large distances over water and lesser distances over land. Studies have found long-range spread across the Mediterranean Sea from North Africa to Spain [13], and from Indonesia and Papua New Guinea to Australia [15], [29], for example. A number of studies have examined long-distance movement of *Culicoides* via a Bluetongue virus (BTV) spread surrogate [13], [14]. These studies have used serological sampling of what are thought to be the index animal cases in previously disease free areas. The date of sampling is known and was used to estimate the date of infection. Using temperature data and experimentally derived data on the BTV incubation period the arrival time of BTV carrying midges has been estimated. Prevailing winds have then been used to determine the source location of the midges. If the location has animals with the same BTV serotype and the only route of BTV spread could have been via transportation by the midge vector (i.e., no animal movements from possible source locations to the arrival location of the virus) then a likely path of midge movement is detected. Data from such studies indicate distances of greater than 100 km over water (and lesser distances over land) are possible, indicating that any modelling of midge or BT spread should include a wind-driver midge dispersal component.

*C. brevitarsis* occupies a particular environmental niche in that it lays its eggs solely in cattle dung [30]. Like other insect vectors, only the female midge bites the mammal host, as it requires blood meals to aid egg development. The population dynamics of both mosquitoes and midges are highly dependent on weather conditions, particularly on temperature. Each species has an optimal temperature range where flying, feeding and mating activity is at its maximum, which also minimizes the egg development period following mating. Temperature also affects the extrinsic incubation period, the time from insect infection (by biting an infected host animal) to becoming infectious itself and thus capable of virus transmission to a new, possibly uninfected, host.

## Methods

### 2.1 Modelling Landscape via Discrete Cells

The aim of the simulation model is to predict the spread and population establishment, growth and die-out of *Culicoides* midges over the landscape through time. This is achieved by representing the *state* of the landscape – that is, landscape information which is relevant to midge spread and population dynamics including habitat-dependent features – in a data structure. A simulation algorithm is then used to capture the *physical processes* which contribute to insect spread using computer software which updates the data structure to reflect the physical system changes, from one simulation time step to the next.

The landscape is represented by dividing it into a regular array of *spatial cells* of similar area, depending on the resolution required. In this study a graticule (i.e. a grid based on lines of latitude and longitude) with 0.05 degree spacing have been used, giving cells that are approximately square with 5 km sides, each with an area of approximately 2500 hectares. The 5 km cell spacing is small enough to represent the spatial heterogeneity in climate (such as altitude dependent temperature) and to represent midge dispersal (as discussed in subsection 2.3 below), while at the same time being large enough to make simulation time tractable.

Single cells are the finest level of spatial detail captured by the simulation model and cells are considered to have uniform characteristics throughout their area. Each cell has a centroid location (latitude/longitude co-ordinates and altitude) and additional information which captures the *state* of the landscape represented by the cell. A summary of the data contained in each landscape cell is given below; further details are given in subsequent sections where the simulation processes that update each cell state are described.

*Geographical data* includes latitude, longitude, altitude and area. The relative location of cells determines distance and direction between neighbouring cells, which influences the spatial dispersal of midges by prevailing wind conditions.

*Weather data* includes daily mean temperature, wind speed, and wind direction. Weather data enters the model as an input data time series. Temperature, including lower temperatures due to altitude, influences the model in multiple ways including the insect reproduction rate, survivability and biting and movement activity. Wind drives vector dispersal.

*Vector habitat data.* For *C. brevitarsis* the key characteristic of the habitat is whether cattle are present or not within each cell, as cattle are necessary for ovipositioning, as *C. brevitarsis* only lay eggs in cattle dung [30], and also for females to blood feed (necessary for egg development within the female).

*Vector population data.* Two population density variables, for the adult and immature stages (egg, larvae, and pupae collectively), represent the *C. brevitarsis* population state of each cell. Unlike geographic, weather or vector habitat data, the vector population data represented in the model are endogenous state variables which both influence, and are influenced by the simulation dynamics.

Cell data fields are given in Text S1, Tables S1.1 and S1.2.

#### Simulation methodology

As the landscape is approximated by a discrete array of cells, the time course of midge spread over the landscape is characterized by local processes that occur within individual cells and processes that model the movement of insects between cells. Time is also treated discretely; state changes (called transitions) occur only at the discrete time points when one time period changes to the next, and the state variables remain fixed for the duration of each period. The dynamic behaviour of each discrete landscape cell is modelled by a corresponding *automaton*, a mathematical device which captures the changing state of a cell based on its current state and the state of neighbouring cells with which it interacts [31]. An automaton consists of state information together with a transition function which models how the state changes through time. This is an *automata theoretic* approach to modelling the inherently continuous behaviour of a complex system; which in this application involves the location of the midges in discrete time and space.

**Figure 1:**
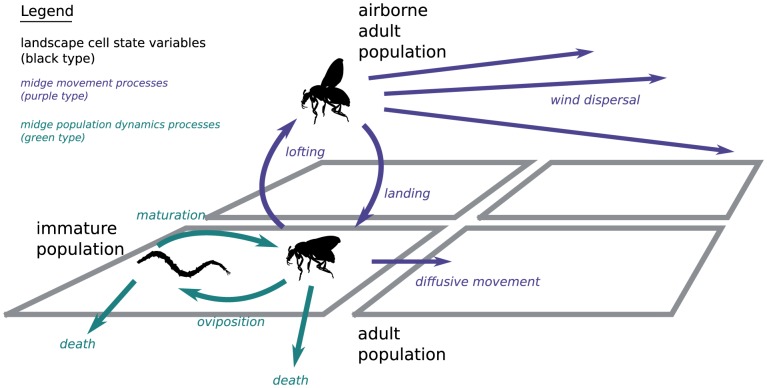
Schematic overview of the simulation model components. The dynamic state variables (population densities) are shown in black type. The processes midge movement modelling midge movement are shown in purple; processes modelling midge population dynamics are shown in green.

This conceptual automata theoretic model is implemented in software and the dynamics of the midge population (involving population growth and decline) together with vector movement is realized by discrete event simulation [32]. The landscape cell automata state data and the transition functions are used by the *simulation algorithm* to update the state of each automaton at an appropriate discrete time step, capturing the dynamic behaviour of the physical system being modelled. An outline of the simulation algorithm is given in Text S1. The specific dynamic processes that determine *Culicoides* spread, namely the changing weather, insect population dynamics, and insect movement between cells may be treated as sub-models which are combined together to produce the overall simulation system. These sub-models are depicted schematically in Figure 1 where 4 discrete cells are pictured for illustrative purposes, and are described in detail in subsequent sections.

#### Model timelines

The main simulation algorithm operates by updating the *Culicoides* population within each landscape cell in a daily cycle. *Culicoides* population dynamics and self-propelled midge movement are calculated on a daily basis, while wind-driven midge movement is calculated on a finer 3-hourly cycle, with 8 such cycles occurring within each daily cycle.

#### Simulation system implementation

The simulation system has been implemented in the Java programming languages and this computer code inputs structured text files containing landscape and weather data sets and scenario descriptions, and outputs text files containing simulation results. These outputs are then processed by a suite of Python-based scripting tools to generate the maps, population time series charts and tabular results presented in this paper. The statistical analyses described in Section 2.4 “Quantifying agreement between simulated and observed midge spread” using Cohen's Kappa were performed using the standard “irr” library of the R software package.

### 2.2 Weather

The distribution of vectors over the landscape and changes in vector density over time is influenced by the weather, specifically temperature and wind speed and direction. This variability is taken into account through the spatial weather model.

#### Temperature Data

Daily maximum and minimum temperatures were obtained from the Australian Bureau of Meteorology for an approximately 5 km square cell grid covering the land area of Australia (including NSW), for the period 1980–2000. This data set is based on automatic weather station (AWS) and topographic (altitude) data and uses a Barnes successive correction technique to interpolate temperature at each grid point [33], [34]. Mean daily temperatures, which are well approximated by the averaged daily maxima and minima, were used as inputs for temperature dependent population dynamics sub-model (described below). Temperature also influences midge flight behaviour with research indicating that *C. brevitarsis* midges take flight when the temperature is 18°C or greater [20]. Days when the mean daily temperature was greater than or equal to 18°C were regarded as “midge activity” days, a concept used by the midge dispersal sub-model (described below).

#### Wind Data

Data from the Australian Bureau of Meteorology for all automatic weather stations (AWS) in NSW for the 1980–2000 period at three-hourly resolution was obtained. Data from 133 AWS were used – a map showing the distribution of weather stations is included in Text S1, Figure S1.2. This wind speed and wind direction data (measured at 10 m above ground) was used in the midge dispersal sub-model. Each landscape cell used wind data from the nearest AWS. Intermittent gaps in AWS data were overcome by taking data from the nearest AWS that had valid data for the gap period.

### 2.3 *Culicoides* Population Dynamics

When significant cattle numbers are present, the key determining factor in *C. brevitarsis* population density is the climate. In areas where the climate is favourable, specific *Culicoides* species may be present and active all year round [35]. In other areas, the *Culicoides* population, and its activity, may become low during winter as a result of lower temperatures. In still other areas, the climate may support incursions of *Culicoides* populations during summer, but extended cold winters may render them locally extinct; this is the case in New South Wales, Australia which is the scenario under consideration here [11], [35].

The characteristics that constitute vector habit vary between *Culicoides* species, however cattle are necessary for *C. brevitarsis*. The rate at which *C. brevitarsis* populations can grow and the maximum density attainable depends critically on the presence of cattle and the availability of dung for ovipositioning (i.e. egg laying) [30], in addition to the temperature. In areas of the landscape being modelled where cattle are absent, such as in National Parks and urban areas, no *C. brevitarsis* population can be sustained and any insects dispersed into these areas fail to establish a breeding population. Any transported midges that come to rest in these cells are assumed to die without having any further effect on the simulation. Note that these cells can carry an airborne population, so these areas do not form a complete barrier to midge spread.

When supplied with an initial insect population and daily temperature time series, the population dynamics sub-model described below generates adult and immature stages of the midge population as a time series for each landscape cell. Temperature-dependent midge population dynamics generated by the simulation sub-model are presented below, in the subsection entitled *Climate scenarios*.

Each landscape cell may be in one of three *insect population states*:

- *Active*; indicating that midges are present in the cell and are actively flying (necessary for feeding and mating) and that the breeding cycle is on-going. In cold conditions, breeding and development rates may slow to the point where they are exceeded by the adult death rate, in which case the population will fall, and may become inactive.
- *Inactive*; indicating that adult population numbers are very low. If temperatures subsequently rise, a cell in the inactive state may become active as immature *Culicoides* emerge from their pupal stage. Alternatively, sufficiently cold and sustained conditions may kill or render unviable all adult and immature *Culicoides*, making the insect population extinct within that cell.
- *Extinct*; indicating that no viable *Culicoides* are present in any life stage (adult, larvae, or pupae). As a result, improving changes in weather or habitat conditions will not cause any change in the cell population state. However, if new midges are transported into the cell and conditions are favourable, the cell state may then transition into an active state. It should be noted that while the active state can be detected from midge trapping programs, the inactive and extinct states cannot be distinguished in this way. The extinct population state may be inferred retrospectively from the lack of trapped midges once the temperature has risen to the point where any active population would be detected.

**Figure 2:**
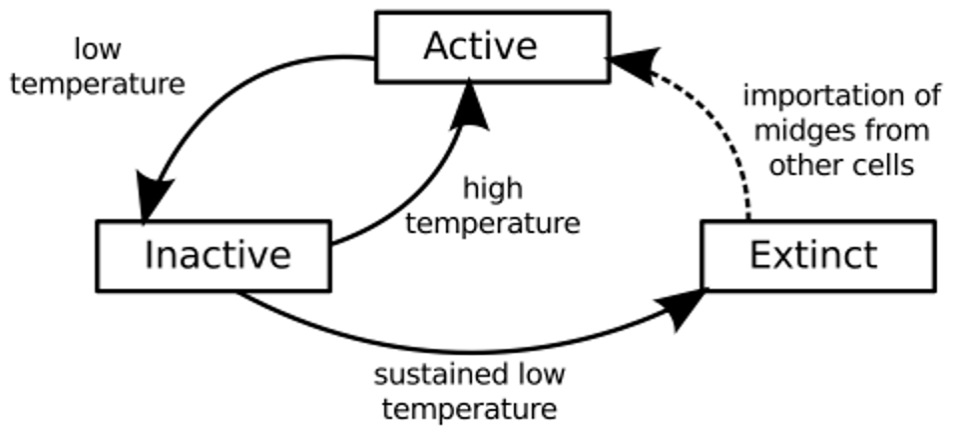
Landscape cell vector population state. Landscape cell population states are pictured as boxes; arrows indicate transitions between states with arrows labelled according to the events or conditions that trigger the state transition.

These states and the possible transitions between them are illustrated in Figure 2.

Cells in the active and inactive states have two additional numeric attributes representing the population density of the adult female (*p_a_*) and immature (*p_i_*) *Culicoides* (taken to include all pre-adult stages) in the landscape cell. We did not attempt to estimate absolute midge population density. Instead, we assume that at any given time the numerical “size” of the simulated midge population is some proportion of the maximum population in the cell. We have set the population scale by defining the (maximum) carrying capacity of the immature population of a cell to have an arbitrary numerical value of 100. Other midge density values that appear in the model are interpreted as relative to this value.

It was assumed that population density evolved according to a logistic population model [36], [37]. This is a standard model of population dynamics that exhibits a maximum growth rate when unconstrained by resources, but where the growth rate decreases with increasing population density, representing increasing competition for some finite resource required for growth. In the model three on-going processes modify the density of the adult and immature populations: the oviposition of eggs by adult females; the maturation of eggs into adults; and the death of both adults and immatures. The rate at which these processes occur is temperature dependent. In the process descriptions below, rates are given for key temperatures, and the dependency of the rate on temperature were modelled as piecewise linear functions of temperature (i.e. rates were interpolated linearly between key temperature values). One particular temperature plays a role in several processes – we refer to this as the *low temperature activity parameter* (LTAP). The LTAP value of 7°C was determined using data distinguishing C. *brevitarsis* climatic zones, as described below in Section 2.4 (in the subsection entitled *Population initialisation*).

**Figure 3:**
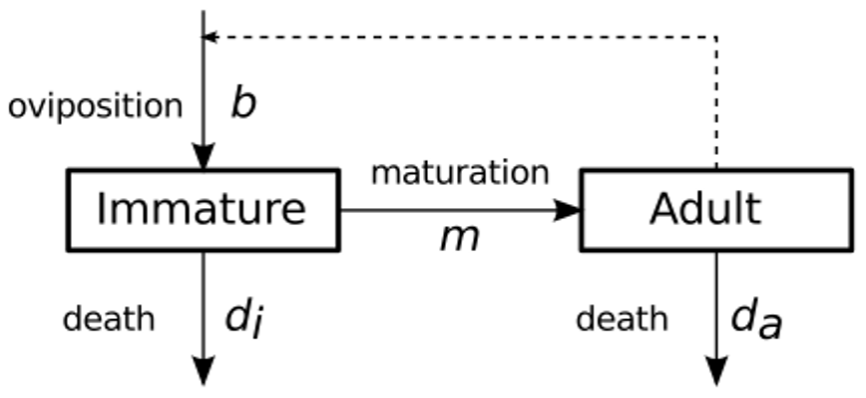
Vector population dynamics sub-model compartments. State transitions of individuals are indicated by solid lines (with the associated rate parameter symbol given in italic type); the influence of adult population on oviposition rate is indicated by the dashed line.

1. *Oviposition*. Adult female *Culicoides* perform a cycle of blood feeding followed by oviposition (egg-laying). The rate at which adult midges lay eggs (denoted by parameter value *b*) in Figure 3 depends on temperature. We assumed (from [12], [38], [39]) that a maximum oviposition rate of 3.9 viable eggs per adult female per day at temperatures of 25°C and above, a rate of 1.1 at a temperature of 18°C, and a rate of zero at the LTAP temperature. These rates were derived by dividing the fecundity (number of eggs layed per oviposition) by the mean gonotrophic period, and further dividing by two (since half of the eggs laid are destined to emerge as males and are not included in our adult population density measure which represents only females [12]). *C. brevitarsis* fecundity averages 31.3 eggs per oviposition [38]. Data on the gonotrophic period and its temperature dependence in *C. brevitarsis* is sparse; we assumed that the gonotropic cycle length varies from a minimum of 4 days to 14 days based on studies of *C. sonorensis* [39]. The temperature point of 25°C giving the maximum oviposition rate corresponds to the temperature of the shortest gonotrophic period reported in [39], while the 18°C value corresponds the minimum temperature at which C. *brevitarsis* have been observed to fly (and thus to oviposit). Rather than assume that oviposition ceases exactly at 18°C, we assume that it decreases gradually to zero at the LTAP value, at 7°C.
  It was assumed that there is a limiting population level for immature midges, which in the case of *C. brevitarsis* is determined by the availability of cattle dung, which provides habitat and nutrition for immature stages; density limited *Culicoides* larval development is reported in [40]. It was assumed that the number of viable eggs laid decreases with increasing immature population density, since eggs laid in already crowded dung will fail to develop due a lack of available nutrition. An alternative method of modelling the immature population density limit would be to increase the immature death rate in crowded conditions; however it is not obvious that this would be a more accurate assumption, since it might be the case that later-laid eggs do not significantly decrease the mortality of more developed larvae (or pupae) originating from earlier laid eggs. As explained previously, an arbitrary value of 100 was chosen for the limiting immature population density, and all other absolute population quantities are relative to this value.
2. *Maturation*. *Culicoides* larvae hatch from eggs and develop into pupae, from which they emerge as adults. Based on experimental data for *C. brevitarsis* [12], the maturation rate (denoted *m* in Figure 3) was assumed to be 11 days at 36°C, 37 days at 18°C, and zero at the LTAP temperature.
3. *Death*. Adult and immature *Culicoides* were assumed to die at a given temperature dependant rate. There are no published estimates of adult lifespan for *C. brevitarsis*: based on data on *C. sonorensis* [39], it was assumed that the mean lifespan varied from 4 days at 25°C, to 14 days at 12°C and below.
  Estimates of immature midge lifespan and dependence of lifespan on temperature were based on experimental data where cattle dung that had been exposed to oviposition was collected and subjected to different temperature treatments [12]. Based on total numbers of adults emerging from dung held at 17°C for 28 and 42 days, it was assumed that immature *C. brevitarsis* had a mean lifespan of 30.5 days. Note that the immature “lifespan” is the mean time when an immature *C. brevitarsis* (egg, larva, or pupa) dies, given that it has not matured into an adult. While total numbers of emerging midges increased with increasing temperature above 17°C (up to 25°C), this might be due to an increased maturation rate (with more *C. brevitarsis* maturing into adults rather than dying in immature stages) rather than increased lifespan. We therefore adopted a simpler model with constant immature lifespan above 17°C. Similarly, the experimental data showed that mortality might increase at lower temperatures; however the numbers of emerging midges were too low to allow quantitative analysis of lower temperature lifespan. Consequently we assumed that immature lifespan decreased to 1 day at the LTAP value.

We note that the use of a single temperature at which oviposition and maturation ceases is a simplification of the actual temperature dependencies; however this model was able to adequately reproduce the observed climatic zones. A more sophisticated model could be substituted if additional *C. brevitarsis* data becomes available.

The relationship between these processes is illustrated in Figure 3. The population dynamics model variables and parameters, along with parameter values and supporting references are summarised in Tables S1.3 and S1.4 in Text S1.

The vector population dynamics sub-model cell can be described as a variant of the classic logistic population dynamics model [36], using two ordinary differential equations (ODE) as follows.
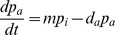


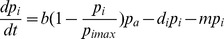



The top equation captures the dynamics of the adult midge population *p_a_* in terms of the maturation rate *m* (of immatures *p_i_* into adults) minus those adults which die *d_a_ p_a_*. The lower equation models the dynamics of the immature midge stages in terms of the birth rate *b* (following oviposition by adult females) which reduces to zero when the habitat capacity of that cell reaches a maximum *p_imax_*. The size of the immature population is depleted by the immature death rate *d_i_ p_i_* and by the maturation of immatures into adults *m p_i_*. Note that the parameters *b*, *d_i_*, *d_a_*, and *m* are functions of temperature, as described previously.

The implemented model differs from the ODE system described above in three ways.

1. The model is a discrete-time difference equation with one-day time steps. In other words, for each cell, the temperature-depended rate parameters are calculated using the temperature for that day; the numbers of ovipositions, maturations, and deaths are calculated using the rate parameters and current populations, assuming the rate maintains a fixed value during the day; and the immature and adult population numbers are then updated accordingly. This process is fully deterministic.
2. When mature and immature populations (a) fall below levels designated minima ε_i_ and ε_a_, respectively and (b) are decreasing, it is assumed that they become zero. Without this feature, vector populations would never become extinct regardless of how close to zero they become, which is unrealistic. Since we did not attempted to estimate absolute midge populations, values of 0.001 and 0.0005 were chosen as small but arbitrary values for ε_i_ and ε_a_ respectively. A sensitivity analysis showed that, ε_i_ and ε_a_ could vary over two orders of magnitude without changing the outcome of the population sub-model calibration process (see Section 2.4 below). The stipulation that small populations only become extinct if they are decreasing has the consequence that when very small midge populations (less than ε_a_) are dispersed into an empty cell, they do not automatically become extinct. Rather, if the temperature in the destination cell is conducive to population growth, a population will become established; otherwise the dispersed population will become extinct.
3. The immature *Culicoides* population density limit factor is not (1−*p_i_*/*p_imax_*) but max (0,1−*p_i_*/*p_imax_*), i.e. when *p_i_*>*p_imax_* the oviposition rate is zero and does not become negative.

#### Significant temperatures

As a result of temperature dependencies, the behaviour of the population dynamics sub-model has two important temperature regimes.

At temperatures above 17°C, adult activity is high, giving a high oviposition rate. Although adult mortality increases with temperature, the oviposition rate also increases meaning that fecundity does not decrease with increasing temperature. In addition, the immature maturation period is short and most immature midges successfully emerge and do not die in the immature state [12]. In this temperature regime, the population grows until the oviposition rate is limited by the immature population density *p_i_*/*p_imax_*. This is the *active* state referred to in Figure 2.

At temperatures below 17°C adult activity is low, giving a low oviposition rate. Furthermore the immature maturation period is long, becoming comparable to the immature lifespan, and immature mortality becomes significant. With fewer midges emerging and a slow rate of oviposition the immature and adult populations decline and eventually become extinct. Note that due to the relatively long immature lifespan, the immature population may take several months to become extinct after the initial adult population collapses. The period between the adult and immature populations becoming zero is the inactive population state referred to in Figure 2.

In addition to these processes, insect dispersal also alters cell population states, with adults being moved out of some cells and into others; see Section 2.3 below.

#### Climate scenarios

**Figure 4:**
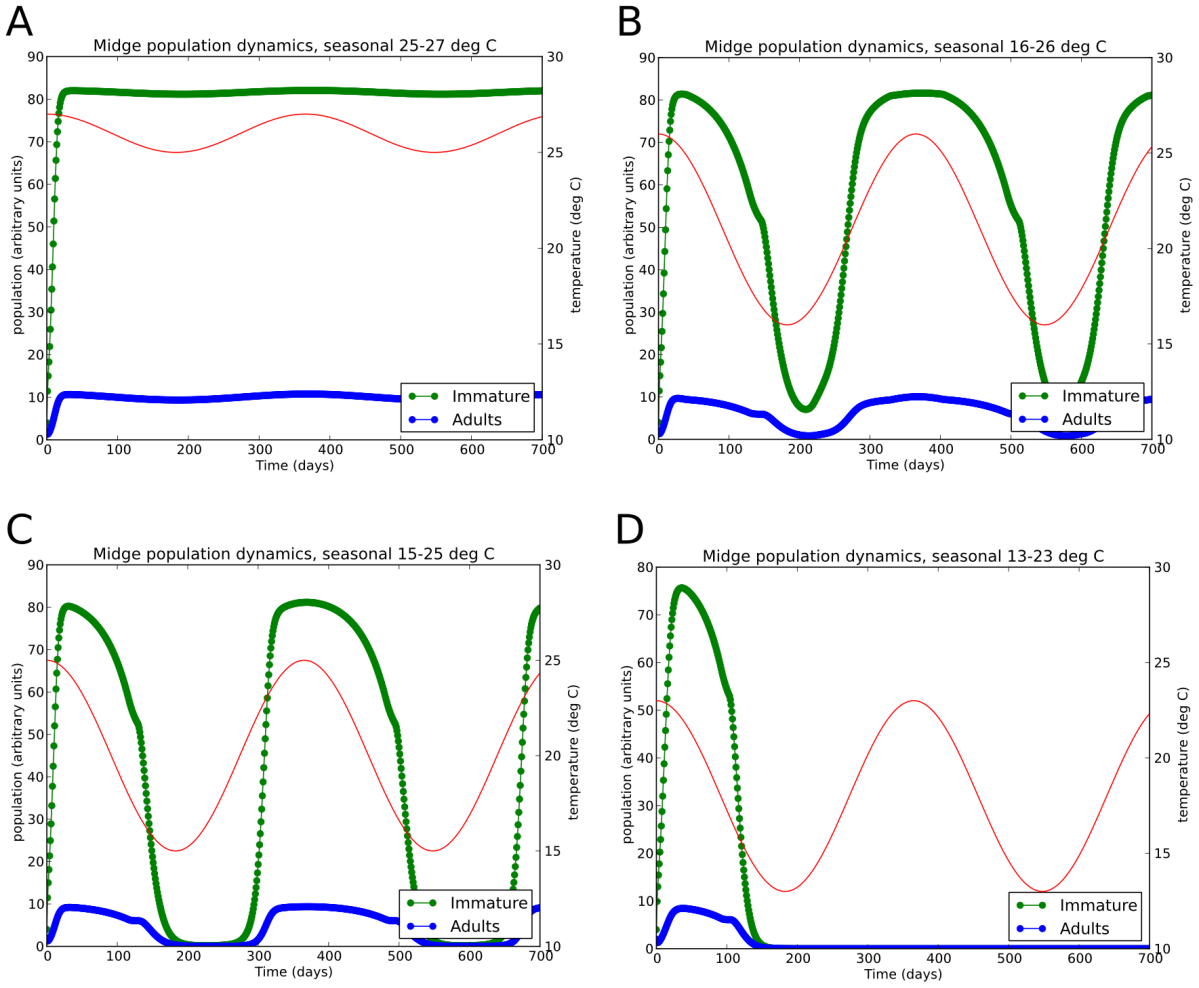
Effect of temperature on simulated midge population dynamics. Daily mean temperatures are shown in red with the scale on the right axis. Population densities are shown in green (immature population) and blue (adult population), with the scale on the left axis. Population density is given in units where the maximum sustainable immature population density has a value of 100. Four idealised climate temperature profiles are shown. A: 25–27°*C*, B: 16–26°*C*, C: 15–25°*C*, D: 13–23°*C*.

The vector population sub-model is capable of representing *Culicoides* population dynamics having three distinct climatically driven patterns, each of which has different consequences for BTV incursion and transmission. Simulations of these climatic scenarios for *C. brevitarsis* are presented in Figure 4, following seeding of midges from day zero.

1. Regions in which the climate allows *C. brevitarsis* populations to exist actively throughout the year. In these areas the midge population remains in the high-temperature regime (above 17°C), although the population may seasonally fluctuate as the activity and breeding rate varies with mean daily temperature [18], [41], [42]. This population dynamics scenario is illustrated in Figures 4A and 4B, which show simulation output of population time series for areas where the mean temperature varies seasonally from 25–27°C and 16–26°C respectively, following initial “seeding” of midges from time zero.
2. Regions in which the midge population undergoes large fluctuations but does not become extinct. In these areas the population is in the high-temperature regime in spring, summer and autumn but falls into the low-temperature regime for a period during winter. There may be times of the year in which adult *C. brevitarsis* population becomes very low (and may also be incapable of transmitting BTV) but the *C. brevitarsis* population recovers each year without external introduction when the temperature rises and surviving immature stages emerge and re-start the breeding cycle [12]. This scenario is illustrated in Figure 4C, which shows simulation output for an area where the mean temperature seasonally varies from 13–21°C.
3. Regions in which *C. brevitarsis* can only survive seasonally. In these areas incursions may result in a population becoming established due to higher summer temperatures, but in winter the population reverts to the low-temperature regime for such a duration that both the adult and immature populations become extinct [11]. This is illustrated in Figure 4D, which has conditions two degrees cooler than Figure 4C. Note that the fact that the number of *C. brevitarsis* found by trapping programs falls to zero does not show population extinction by itself. The inference that the population does become locally extinct is made from fact that when the temperature rises the following spring, trapped midge numbers do not rise with the rising temperatures as they do in warmer northern areas where they do clearly overwinter. Instead, midges are not detected until a time delay has passed, with the delay being approximately proportional to the distance from *C. brevitarsis* endemic areas [43].

The model of *Culicoides* population dynamics described here is based on *C. brevitarsis* but model parameterisation allows for the population dynamics of other *Culicoides* midge species to be represented.

### 2.4 *Culicoides* Midge Movement

Two types of insect movement can occur, *wind-blown dispersal* and *diffusive spread* via active flight. Each cell centroid location (latitude/longitude co-ordinates and altitude) and cell size can be adjusted to suit the scale of the simulation, as a trade-off between spatial resolution and computational efficiency. The relative location of cell centroids determines the distance and direction between neighbouring cells, with between-cell dispersal of midges depending upon the distance between cells and prevailing wind direction and speed.

#### Diffusive spread

*Culicoides* midges are significantly smaller than most mosquito species and do not exhibit self-propelled long range movements. In the absence of wind or other directional stimuli, they can be assumed to move according to a random walk process with typical movement ranges up to 100 m per day. Such behaviour has been observed by trap-mark-release-trap field experiments which showed that typical daily (or nightly) flight ranges of midges are mostly on the order of a few hundred meters [44], [45]. The collective movement behaviour of a large number of insects executing a random walk can be modelled as a diffusion process [46], [47]. When implemented in our cell-based spatially discrete simulator, this process moves a small proportion of midges to neighbouring cells during each simulation cycle. The same trap-mark-release-trap experiment cited above showed that at least a small percentage of midges travelled at least 4 km in 24 hours, so it is expected that a small percentage of midges will move to a neighbouring 5 km cell in each 24 hour simulation cycle, and our representation of self-propelled midge movement as a diffusion process captures this phenomena.

In a cellular implementation of a diffusion model, the quantity of particles (in this case midges) moving between cells depends upon the population density of the cells, the cell size, and a diffusion coefficient which characterises the movement of the diffusing species. Two studies report diffusion coefficients for *Culicoides* species: 60.1 m^2^/s for *C. impunctatus* [47], [48] and 12.96 m^2^/s for *C. variipennis* [45], [49]; there does not appear to be any similar quantified dispersal data for *C. brevitarsis*. The *C. variipennis* value was adopted, since the methodology by which it was derived is described in much greater detail compared to the *impunctatus* value. A sensitivity analysis was performed which examined alternative diffusion coefficients ranging from 6.5 m^2^/s to 120 m^2^/s (see Text S1). Results of idealised proof-of-concept simulations demonstrating the effect of the diffusive midge transport model are shown in Text S1, Figure S1.1.

As indicated by experimental data on C. *brevitarsis* behaviour, diffusive dispersal was assumed to occur only on days when the mean temperature was 18°C or greater. Further details of the implementation of the diffusive movement model can be found in Text S1.

#### Wind-borne dispersal

For the purposes of wind dispersal, each 1-day simulation cycle was also broken in to 3-hour wind sub-cycles. This fine grain time period is required as the flying behaviour of most midge species differs between dawn and dusk, and other times of the day. *Culicoides* are active (that is fly, feed, mate and egg-lay) when winds are no stronger than 8 km/h. If winds are stronger they generally stay on the ground attached to plants [20]. *C. brevitarsis* are known to be most active immediately before and after dusk and active to a lesser extend just before dawn. Once flying they may be lofted above their usual 3–4 meter flying height by thermals or by topography-induced wind turbulence, allowing them to reach higher altitudes with possibly stronger winds [13], [15].

Wind-driven midge transport was modelled representing two midge sub-populations in each cell, a “grounded” and a “flying” population. It was assumed that three processes occurred in each landscape cell during each 3-hour period:

1. *Lofting*. Based on observations of *C. brevitarsis* flight behaviour, it was assumed that grounded adult (female) midges take to the air on days when the mean temperature was 18°C or greater, during periods approximating midge activity around dawn and dusk (6pm to midnight, and 3am to 9am), but only when the wind speed was less than, or equal to, 8 km/h [20]. The rate that midges became airborne was such that on average half of the midges in a cell would become airborne in 24 hours of continuous favourable flight conditions (we assume that lofting is a Poisson process [50], which translates to 6% of midges becoming airborne in each favourable 3-hour period). There is unfortunately no existing data to inform this model parameter. A sensitivity analysis was conducted to assess the sensitivity of the overall spread dynamics to this parameter (details can be found in Text S1, Table S1.6).
2. *Wind transport*. Midges currently airborne were transported into a number of cells in a “footprint” downwind of the source cell. It was assumed that midges would be carried at the speed of the wind. The AWS wind data source used recorded winds at a standard height 10 m above ground. We considered that midges may be transported by winds which may be faster or slower than 10 m winds, since wind speeds generally increase with altitude due to the surface wind gradient. We assumed that the maximum midge transport speed was some multiplicative factor of the recorded 10 m wind speed (as midges may be carried at higher altitudes), and calibrated this multiplier to achieve the best fit to observed *C. brevitarsis* arrival times at trapping sites in NSW in 1991/1992 [11]. This calibration process is described below in Section 2.4 (in the subsection entitled “Wind transport sub-model calibration”). It was found that multiplying the 10 m wind speed by a factor of 4 provided the best match to the dispersal data (further detail can be found in Text S1, Table S1.5). The footprint used was a wedge shape; specifically, a circular sector with radius given by the spread speed multiplied by the 3-hour wind dispersal cycle duration and subtending an angle of 60 degrees, representing fluctuations around the average wind direction reported in the AWS weather data. Wind transported midges were distributed evenly into the airborne population of all cells in the dispersal footprint including the source cell. It should be noted that using this mechanism, the use of large cell spacings will artificially truncate wind dispersal at low wind speeds, when the maximum dispersal range is less than the cell spacing. Our choice of grid cell spacing of 5 km with a 3-hour wind dispersal cycle period is sufficiently small that this problem is avoided.
3. *Landing*. Currently airborne midges were assumed to land at the same rate at which they became airborne. Like the lofting rate, there is no data available to inform this parameter value, however sensitivity analyses showed that overall spread dynamics were insensitive to this parameter (see Text S1, Table S1.7 for further details).

Note that this dispersal mechanism allows wind-driven transportation at speeds higher than the “take-off” threshold, since it allows midges to become airborne and stay airborne even if the wind speed subsequently increases beyond the 8 km/h threshold. The model of midge dispersal used here is based on field studies of *C. brevitarsis* flying behaviour [20] and long-distance dispersal [11], [43], however the model is parameterised so that flying behaviour of other midge species can be readily represented. Results of idealised proof-of-concept simulations demonstrating the effect of the wind-borne midge transport model are shown in Text S1, Figure S1.1.

### 2.5 *Culicoides* Spread Simulation Experiments

#### Population initialisation

**Figure 5:**
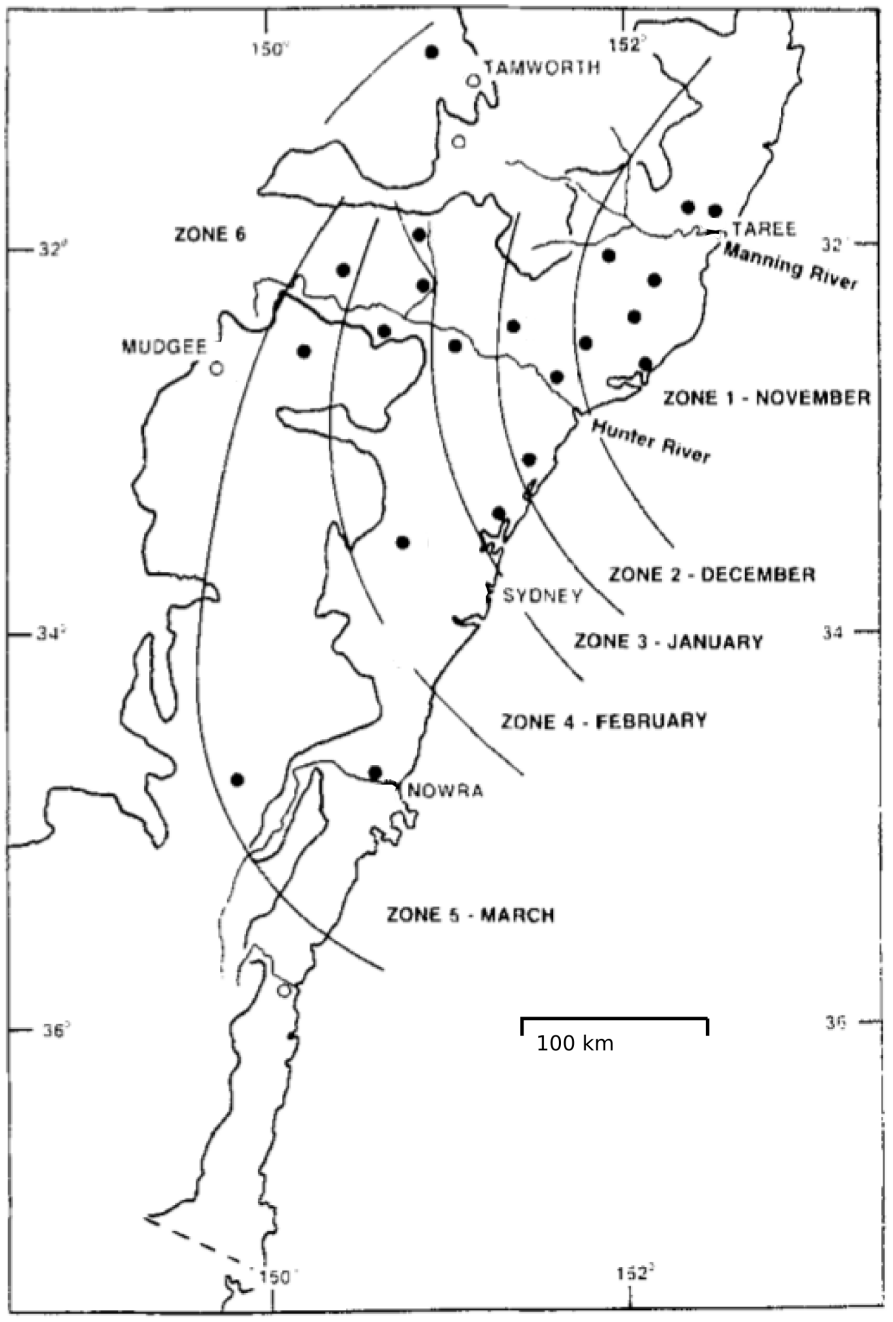
*C. brevitarsis* spread arrival times as determined by trapping experiments. Lines denote arrival times of *C. brevitarsis* derived from trapping data in New South Wales in 1991/2, classified by monthly zones. This figure is based on Figure 2 from [11]. Circles denote trapping site locations; filled circles indicate sites at which *C. brevitarsis* were detected, open circles where not detection occurred.

The northern coastal area of the region being considered in this study is pictured in the north-east corner of the map shown in Figure 5 and contains an endemic population of *C. brevitarsis* [11]. A viable population is known to persist throughout the mild winter period and subsequently increases as the weather warms during spring and summer [18]. This area is the source of midges which then spread southwards as the season progresses, as reported in the following [11], [16]–[19]. The area containing endemic midge populations was initialised by the simulation model using the population dynamics sub-model as follows.

All cells in the region were seeded with adult and immature stages equally and the population dynamics process was run without any midge dispersal between cells. This simulation used 1-year temperature series for each cell (provided by the Australian Bureau of Meteorology), starting on 1^st^ January 1991, which is summer in the Southern Hemisphere; 1991 was selected as this was the year preceding the year when most *C. brevitarsis* trapping occurred [11]. Consecutive years were used so that the temperature data series ran continuously from the year used for population initialization (1991) through the year used as the primary comparison between simulated and observed midge spread (1992/3). This initialization procedure allowed for temperature differences between northern and southern areas of the modelled region to impact on population growth and, importantly, subsequent extinction. The low temperature activity parameter (LTAP) in the population dynamics sub-model was adjusted between successive applications of the initialization procedure until immature stage midges became locally extinct (in winter) in the same areas as reported in the literature [11], [16], [18], [19] as being too cold to support overwintering. Specifically, the LTAP was adjusted in 1°C increments until a dividing line between the northerly overwintering population and southerly population (which became extinct) occurred south of the Hastings valley (31°S) but north of the Manning valley (31.9°S), see Figure 5. It should be noted that the LTAP value derived by the calibration process also depended on the value selected for the parameters ε_i_ and ε_a_ (described previously in Section 2.2). However, while the overwintering behavior depended strongly on the LTAP with small (1°C) changes causing large differences in the overwintering area, it depended only weakly on ε_i_ and ε_a_: overwintering areas were similar for ε_i_ and ε_a_ values spanning two orders of magnitude.

The adult and immature population densities for each cell were recorded at the 1^st^ October 1991 simulation cycle, and this population density map formed the initial conditions for the main simulations described in the results section. Video S1 shows an animated map of the population density during the calibration simulation. In performing this calibration procedure, it was noticed that the geographical occurrence of overwintering and occurrence is very finely balanced. Small changes in LTAP (or in the actual temperature times series) led to overwintering in the lower Hunter valley (see Figure 5), which is consistent with the fact that overwintering is intermittently observed in some years but not others [17].

#### Wind transport sub-model calibration

A series of simulations were conducted to determine the maximum wind transport speed parameter, which accounts for midges possibly being transported by winds at high altitudes and higher speeds than the AWS recorded wind speeds (see Section 2.3 “Wind-borne dispersal”). The population was initialized for 1^st^ October 1991 as described above, and multiple simulations were run varying the wind speed transport multiplier over the range [0.0,5.0] in increments of 1.0. A value of 4.0 maximized the agreement between simulated and observed spread (as described below), and this value was used in subsequent spread experiments (further information can be found in Text S1, Table S1.5).

#### Simulation experiments

Experiments were conducted using the model in the coastal area of NSW bounded by: 31.5 degrees south to 35 degrees south and 149 degrees east to 153.75 degrees east. The area was divided into a grid with 0.05 degree spacing (approximately 5 km), giving 12,350 individual cells. Cells with centroids in the ocean, urban areas of Sydney, Wollongong and Newcastle and national parks were marked as unsuitable habitats due to the need for cattle dung for ovipostioning (egg-laying). All other cells were deemed uniformly suitable *C. brevitarsis* habitats, assuming adequate cattle numbers to sustain the breeding cycle. More detailed cattle density heterogeneity data will be sourced when these modelling techniques are applied to BTV transmission in this region. Unless otherwise stated, simulations were run for 12 months from the beginning of October 1991, when midge populations begin to become active following winter.

#### Quantifying agreement between simulated and observed midge spread

We used a *C. brevitarsis* trapping data set that recorded the month during which *C. brevitarsis* was first detected at various sites in NSW in the summers of 1990/91, 1991/92 and 1992/93 [11]. In the publication describing the data set monthly time periods were reported, using data aggregated from weekly trapping time series data, which exhibited considerable noise at the weekly level. The output of each simulation run included the adult population density for each cell on each simulation day. For each of the trapping sites in the data set we determined the cell that contained the site, and determined the date on which the population rose above a trapping detection threshold parameter, which represented the minimum population density at which C. *brevitarsis* would be detected. Since the relationship between numbers of midges caught in traps and the true midge population is unknown, we selected a small but arbitrary value of 0.05 (i.e. 1/50,000 of the immature population carrying capacity) for the trapping detection threshold. Because simulated midge arrival times depend both on the parameters of the transport sub-model and the trapping detection threshold, it is conceivable that the calibration of the transport sub-model is thus merely reflecting the (arbitrary) choice of the trapping threshold. A sensitivity analysis showed this is not the case: the best-fitting wind speed multiplier parameter was found to have the same value (4.0) for trapping detection thresholds varying over at least two orders of magnitude (in the range [0.005,0.5]). Results of this sensitivity analysis can be found in Text S1. The simulated and observed months of first arrival were treated as discrete variables, and Cohen's kappa statistic [51]. Cohen's kappa ranges from 1.0 indicating perfect agreement to 0.0 indicating a level of agreement expected by chance alone; with negative values indicating systematic disagreement. For each data point (which in our case is an arrival time at a trapping site), kappa penalises disagreements between observed and simulated category values (which in our case are arrival months). The calculation of kappa allows these penalties to be weighted according the magnitude of the disagreement - we weighted disagreements by the square of the number of months difference between observed and simulated arrival. For example, a site at which simulated and observed arrival different by 2 months (e.g. December and February) was penalised four times more than a site with a 1-month disagreement (e.g. December and January). The significance value p is the probability of observing the level of agreement (kappa value) assuming that the true agreement is zero.

## Results

### 3.1 Overview

Results presented in Section 3.2 illustrate the population dynamics generated by the model using seasonally varying temperatures. These highlight the effect which temperature has on *C. brevitarsis* population expansion and decline, as the temperature increases and then falls from spring through summer and into winter.

In Section 3.3, the effect of the two sub-models, viz. midge population dynamics and midge dispersal, is shown to capture seasonal midge incursions moving southwards along the NSW coast. If diffusive midge dispersal occurs without the addition of wind-driven dispersal, the simulated incursions fail to reproduce the rate of southerly spread observed by the field trapping programme [11], [16], [18], [19]. With wind-dispersal added, monthly patterns for midge arrival times at the different trapping locations in NSW are (approximately) reproduced by the model. These simulated data confirm that both sub-models operating together replicate observed *C. brevitarsis* characteristics, that is, the dispersal of midges into “virgin territory”, the subsequent temperature-dependent population establishment and growth in these areas, followed by decline and then extinction.

### 3.2 Population Dynamics

Using actual daily temperature data, the population dynamics sub-model was shown to be consistent with the *C. brevitarsis* population dynamics data in the given locations and reported in [11], [16], [18], [19], as follows.

**Figure 6:**
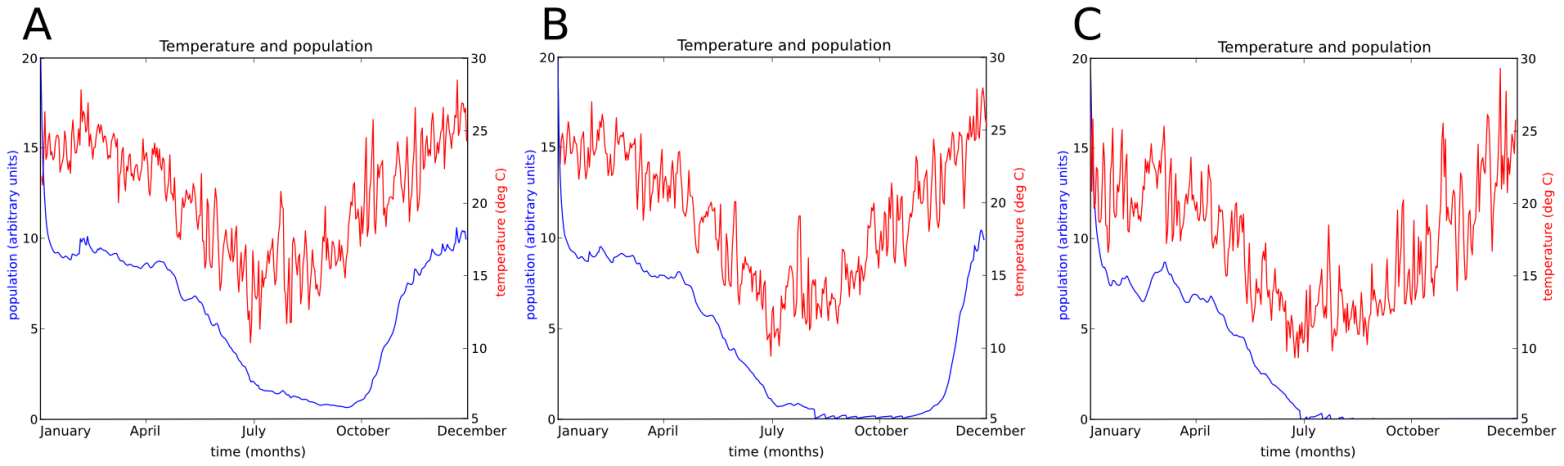
Temperature and simulated *C. brevitarsis* populations. Daily mean temperatures are shown in red with scale on right axis. Adult midge population density shown in blue with scale on left axis. Population density is given in units where the maximum sustainable immature population density has a value of 100. Three time series were extracted from the population initialisation simulation (see Section 2.4 subsection “Population initialisation”) for locations A: Byron Bay (latitude 28.64S), B: Kempsey (latitude 31.08S), and C: Nowra (latitude 34.94S).

Endemic populations where adults are present year-round can occur near the northern NSW state border, for example Byron Bay (latitude 28.64 south). Figure 6A shows the daily mean temperature and simulated population curves for the Byron Bay location over one year starting in January 1990. The data shown in Figures 6A, 6B and 6C were obtained by locating the simulation cell containing the site, and extracting the temperature and adult population density daily time series for that specific cell from the simulation used to establish the initial population (see Section 2.4 “Population initialisation”). The simulated population curve shows that temperature and breeding activity are at a maximum in January and February, explicitly reflecting known temperature dependent population dynamics. The midge population grows during this period and peaks at the end of February. The population then slowly declines with declining temperatures, as the rate of newly emerging midges does not keep pace with the midge mortality rate. Temperature and breeding activity reaches a minimum in August. By the end of October the population begins to increase as the larvae from the increased breeding activity begin to emerge. The relationship between temperature (red) and the adult population (blue) can be seen clearly in Figure 6A.

Further south are areas in which the adult population disappears (i.e. falls below levels where it is detectable by a trapping program) during winter but where larvae survive and quickly re-establish adult populations once the temperature increases. Figure 6B shows temperature and population curves for Kempsey (latitude 31.08 south) which is approximately 350 km south of Byron Bay. In this location the simulated population curve during summer and autumn is similar to that described in Figure 6A except that the population fluctuation is larger due to the greater seasonal temperature variation. At the coldest part of winter the adult population falls to zero: all adults die due to the low temperature and additionally no larvae emerge. Once the temperature increases however, surviving larvae emerge and establish a breeding cycle once more.

Further southwards “down” the NSW coast are areas in which imported populations can survive during summer and autumn but where longer winter cold periods render both the adult and immature populations extinct. Figure 6C shows temperature and population curves at Nowra (latitude 34.94 south, on the southern coast in Figure 5) which is approximately 570 km south of Kempsey. In this simulation a population is assumed to be present at the beginning of the year, due to movement from further north. The population grows in summer, declines in autumn, and the adult population becomes zero during winter. Somewhat later, the immature population (not shown) also becomes zero.

We note that midge populations reported in the literature from trapping programmes [18], [20] are considerably more ‘noisy’ than the simulated population curves appearing above. We believe that this is primarily due to the relative spatial resolution of the simulation model compared to the area sampled by the traps. While the simulation model represents average midge density over a 5 km cell, each trap samples an area approximately 100 m wide, and so highly localised effects come into play, such as daily and weekly movement of cattle into and out of the area near the trap site.

### 3.3 *Culicoides* Spread and Population Dynamics

Midge trapping studies have documented the seasonal spread of *C. brevitarsis* in NSW e.g. [11], [16], [18], [19]. Typically, midge populations are detected in the Manning Valley coastal area in November (see Zone 1, Figure 5), having spread from the endemic areas further to the north. In the following months C. *brevitarsis* midges are successively detected at increasing distances southwards from the Manning Valley. Although prevailing winds are not predominantly from the north in this area, simulations show that periods of north or north-easterly winds are frequent enough to generate “spread events” that distribute *C. brevitarsis* into previously unoccupied territory, from which populations build and spread further. Winds in other directions also transport midges to the north and east; however these transport events do not impact the overall spread of *C. brevitarsis*, as midges are spread back into areas they already occupy, or out to sea where there is no supporting habitat. The distance of southern spread varies from year to year due to weather variability, which includes the number and timing of significant southerly wind spread events, but the overall pattern remains similar; Figure 5 (based on Figure 2 from [11]) shows this spread during 1991/92.

**Figure 7:**
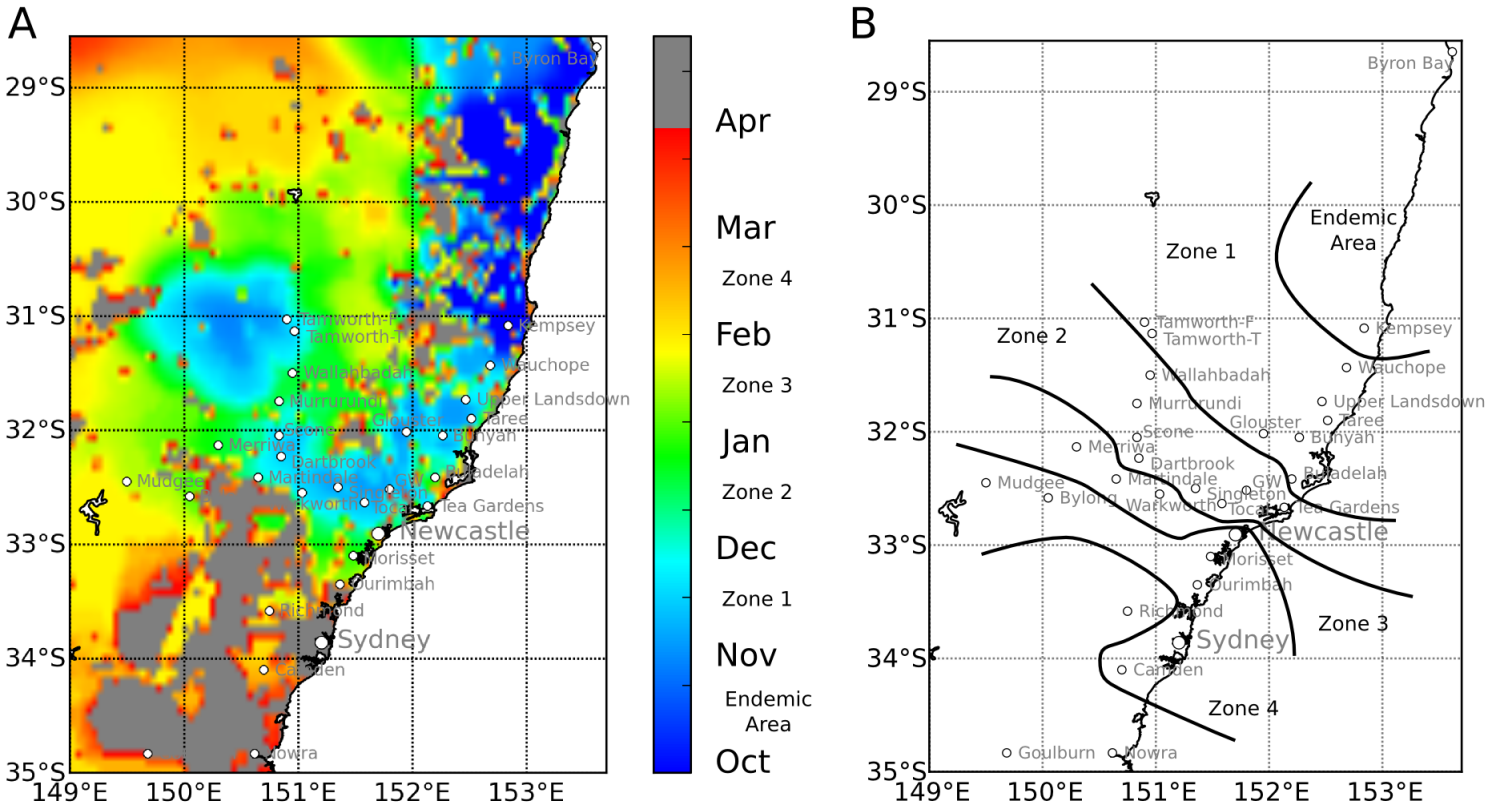
Simulated arrival times following midge dispersal and population establishment. A: Midge arrival times shown by colour for the months following 1^st^ October. White (ocean) and grey indicates areas in which no midge population became established. B: Contours showing monthly arrival time Zones: Zone 1 November, Zone 2 December, Zone 3 January and Zone 4 February.

The combined *Culicoides* population dynamics and dispersal sub-models described in the Methods section was used in a simulation of *C. brevitarsis* dispersal and shown to replicate this southerly spread. The model used actual, spatially-explicit weather data for the 12 months from 1^st^ October 1991. The simulated arrival times when the first *C. brevitarsis* appear are pictured in Figure 7A and by the monthly arrival “zones” overlaid onto the map in Figure 7B. Figure 7B allows comparison with Figure 5, which maps similar monthly arrival time isochrones but which are based on an extensive trapping program [5] (the zone boundaries in the right frame of Figure 7B were drawn by hand to visually separate the sites that fall into each zone). Table 1 presents monthly simulated arrival times together with the corresponding observed trapping months, allowing simulated and actual arrival times to be compared for all locations where trapping occurred, along with a quantitative measure of agreement (Cohen's kappa – see Section 2.4 “Quantifying agreement between simulated and observed midge spread”). Table 1 also includes observed versus simulated midge arrival time comparisons for the seasons immediately prior to 1991/92, namely 1990/91 and 1992/93. Data from these additional years was not used in the calibration of the model, and thus serves as a proof-of-concept validation of the model.

**Table 1:** Simulated and Observed Arrival Times.

	1990/91	1991/92	1992/93
Number of sites (n)	25	24	13
agreement (kappa)	0.468	0.527	0.571
Significance (p)*	0.00205	0.00485	0.00661
**Site**	**observed/simulated arrival month**		
Buladelah	1/3	1/1	2/4
Bunyah	1/3	1/1	2/3
Bylong	5/6	5/3	-
Camden	4/6	4/4	-
Dartbrook	4/4	4/1	-
Glenwilliam	2/4	1/1	3/3
Glouster	1/3	1/1	3/3
Goulburn	5/6	5/6	-
Martindale	3/4	4/2	-
Merriwa	5/3	5/2	-
Morisset	2/6	2/3	3/4
Mudgee	6/6	-	-
Murrurundi	4/4	4/2	-
Nowra	4/6	5/6	-
Ourimbah	3/6	3/3	4/4
Richmond	4/6	4/6	5/6
Scone	4/4	2/1	-
Singleton	2/4	2/1	3/3
Tamworth-F	-	6/1	-
Tamworth-T	-	-	-
Taree	1/2	1/1	1/3
Tea Gardens	1/4	1/1	2/4
Tocal	2/4	2/1	3/3
Upper Landsdown	1/2	1/1	1/2
Wallahbadah	4/3	-	-
Warkworth	4/4	3/1	-
Wauchope	1/1	1/1	1/2

Numbers given for observed and simulated C. brevitarsis arrival times denote the month after October i.e. Zone 1 November, Zone 2 December, Zone 3 January, Zone 4 February, Zone 6 March. A dash (-) indicates that *C. brevitarsis* were not observed (in reality or in simulations) in that year at that location; these sites were excluded from the analysis for that year.

* Significance p-value is the probability that the agreement kappa value would be found given that simulation and observation were uncorrelated i.e. if simulation results were random.

A short animation of the simulated population dynamics and midge spread is provided in Video S2. The animation shows several clear wind transport events where midges are dispersed from locations with established populations to new areas, which then experience their own population growth and onward dispersal into previously midge-free areas. The animation also shows the seasonal cycle of the incursive midge population, with a growth phase in summer and autumn, followed by disappearance of midges during the winter months, first from higher altitude inland areas which experience lower temperatures first, and then from the coastal area, starting from the south and proceeding northwards. New populations grow again in spring from overwintering populations in northerly, but not southerly areas.

### 3.4 Sensitivity analyses

In addition to the main experiments comparing observed midge spread to that generated by the simulation model, we also performed additional experiments to support the claim that a model combining temperature-dependent population dynamics and wind-borne dispersal is necessary to represent the seasonal incursive *C. brevitarsis* population in NSW. Quantitative results of these experiments can be found in Text S1, Tables S1.8, S1.9 and S1.10.

In the C. *brevitarsis* literature it is reported that the low winter temperatures in southern NSW prevent overwintering, and that when midges are detected in those locations this is a result of midges being transported from the north. As an initial test of our model, we considered an alternative hypothesis that midges overwinter in *all locations where they are detected*, and the apparent progression of midges from north to south is actually due to midges being detected at progressively later times due to slower population growth in southerly regions, reflecting temperature rises occurring later in the spring in the southern regions. This hypothesis was examined by running simulations without the midge transport sub-model, using a range of values for the LTAP value, which determines the locations in which overwintering will occur. We then compared the first simulated detection times of midge populations which grow after overwintering, with the first detection times from the trapping data. We found that these simulation scenarios (with overwintering in all locations but without midge transport) could not reproduce the observed midge detection patterns. See Text S1, Table S1.8.

We next conducted simulations to determine if midge spread could be modelled as a purely diffusive local spread phenomena, without a wind-driven component. We found that in order to approximate the observed midge spread, the diffusion coefficient (representing the short-range, self-propelled flight behavior of midges) would have to be larger than any reported value for *Culicoides* midges, such as presented in [45], [47]. See Text S1, Table S1.9.

Finally, we examined how the temperature-dependent nature of the population dynamics affected the timing of midge spread. We found that a model using constant temperature could replicate the observed midge spread, however this model was inferior in several ways. Firstly, it is artificially sensitive to the starting date of the simulation, since population growth and spread occur from the beginning of the simulation; by contrast, the temperature dependent model is insensitive to the simulation start date, since spread begins only when temperatures allow population build-up in newly colonised areas. Secondly, while initial arrival times can be approximated with a constant temperature model, the final state of the midge population at the end of the simulation is very inaccurate, as with constant temperature there is no winter die-off of midges and midge activity occurs all through the year. This precludes the use of a constant temperature for multi-year simulations. See Text S1, Table S1.10.

## Discussion

Models of insect vector population dynamics and movement are necessary when developing biologically plausible models of insect disseminated disease spread. The *C. brevitarsis* model presented here will be used in the future development of a BTV spread model and used to determine the effectiveness of interventions (e.g. vaccination, culling and/or movement bans) in achieving disease free status following an incursion into a previously disease free area [24]. Furthermore, the effects of longer term temperature changes, such as those caused by global warming are captured through the temperature dependencies included in the model. Changes in temperature ranges will potentially change the population dynamics and over-wintering of *C. brevitarsis* in this region. The research challenge is to develop a modelling framework which supports the construction of realistic models which capture the fundamental features of the underlying physical system and, if possible, validate it using field-collected data, as is done in this study for the years 1990/91 and 1992/3 in Table 1.

The model described in this study was sufficiently realistic to permit us to address qualitative questions as to the nature of midge population growth and spread: that establishment of midge populations in southern areas in summer is inconsistent with an overwintering thesis but consistent with midges being transported from endemic areas in the north; and that this involves wind-blown midge transport and not solely self-propelled midge movement. The data in Table 1 indicate that simulated arrival times follow the same pattern appearing in the trapping data, with spread southward and westward from the endemic area as the months progress. However, midge arrival time predictions based on inferred initial populations and observed weather are not especially accurate, having Cohen's kappa 0.527 (p = 0.00485). The major discrepancy between the simulated and observed spread is that in the simulation spread occurs rapidly to the west, over the Great Dividing Range to the NSW Tablelands, from where it spreads south, arriving all along the length of the Hunter Valley at the same time. The trapping data indicates that actual spread does not rapidly cross the Great Dividing Range, but occurs first southerly to the lower Hunter Valley area, and the progressively west up the valley. Limitations of the current model include the omission of rainfall, humidity and variable cattle density, all of which are lower in NSW inland areas, all which may contribute to this inaccuracy. These data-related issues could be addressed in future model developments.

The modelling mechanisms described above have been shown to model the key phenomena which underlie the spread of pathogen-carrying insect vectors and thus spread of the pathogens themselves. With appropriate adaptations these methods have application beyond the *Culicoides* vector and Bluetongue virus, with possible application to human viruses spread by mosquitoes, such as dengue, chikungunya, and Japanese encephalitis [52], [53]. They also have direct application to incursions of mosquito species into new territory, such as the recent spread of the Asian tiger mosquito *Aedes albopictus*, a competent vector for dengue and chikungunya, in southern Europe [54] and Australasia [55].

### 4.1 Related Research

Models of Bluetongue virus generally exclude inherently spatial landscape and habitat features which impact on *Culicoides* population dynamics and movement, such as presented here. For example, the BTV transmission models presented in [26], [27], [56] rely on inter-farm BTV transmission being modelled by a distance kernel which implicitly includes vector dispersal and animal movement. The transmission model for between-farm spread required data from the 2006 northern European outbreak to estimate parameters; no location-explicit *Culicoides* vector data was used and BTV outbreaks in different environments, such as different farm structures, insect vector habitats and climate regimes may not be directly modelled using this approach. BTV spread models with implicit vector populations do not allow for explicit modelling of the spatial spread of BTV via wind-moderated midge dispersal, nor BTV spread in areas where the presence of a *Culicoides* population varies seasonally or from year to year.

Models which explicitly represent the effect of wind speed and direction on BTV spread via midge dispersal have been developed, in the study reported in [28] for example, where the spread of BTV cases is taken to be a surrogate for insect movement. In that model a more sophisticated wind model which included the effects of topography was utilized, compared to that adopted in this study. BTV outbreak data from southern France was used for model parameterization and no model of insect population dynamics was required, as BTV spread into areas with already-present competent *Culicoides* species, in contrast to the vector incursion scenario presented here.

Incursion of arboviruses such as Bluetongue (BTV) and Akabane [11] (also spread by C. *brevitarsis*) into areas without an extant competent vector population do not behave in the same way as incursions into areas with endemic competent vector populations. In NSW, Australia (the same area as used in this modelling study) Murray showed that the arrival of the Akabane virus was delayed 4–6 months relative to arrival of the C.*brevitarsis* vector [11]. By contrast, in the northern European BTV outbreak in 2006 and subsequent years, BTV spread much faster than that observed by Akabane virus spread in NSW. This was a consequence of infected *Culicoides* species moving into areas already containing similar midge vector populations, hence rapidly producing localized outbreaks without the need for populations to become established and the consequent time delay required to raise the vector population to support virus transmission [24], [57]. The NSW scenario presented here required establishment of significant vector populations, causing a delay before virus transmission to/from cattle may occur. By contrast, in the northern European setting a resident, competent population of (paleo-arctic) *Culicoides* species existed and these rapidly became infected once infected midges arrived and infected host cattle (and sheep to a lesser extent) [58]. In the European context, higher host densities and smaller distances between farms may also contribute to the rapidity of BTV spread. Detailed analysis of the 2006 northern European BTV outbreak [59] suggests that observed long-distance BTV spread (over land) may be due to sequences of fairly short (<5 km) ‘stepping stone’ infections rather than long range jumps; a feature which is readily modelled using the fine-grained spatial methods detailed in this paper. The modelling mechanism presented reflects the physical system where populations of midges are dispersed relatively short distances, then establish themselves and take time to increase the size of the local populations in the new regions. Again, this differs from the European setting reported in [59] where populations already exist in the locations where BTV-infected *Culicoides* vectors move into, as a travelling wave of BTV-infection in host animals. In the model presented here we also describe a travelling wave, but in this instance it is of the arrival of the insect vector itself. In order to model Bluetongue incursions in locations where competent vectors are not endemic but are only seasonally present, modelling the invasion of the vector population, as presented here, is necessary to modelling BTV spread itself.

### 4.2 Limitations and further research

While the temperature-dependent population dynamics sub-model does reproduce the different C.*brevitarsis* climatic zones in NSW and is consistent with experimental data on immature midge survival, the role of the climate data was to calibrate rather than validate the population dynamics sub-model. Quantitative validation of the population dynamics model would require year-round trapping data from field experiments at multiple sites in each climatic zone, data which to the best of our knowledge does not exist. Similarly, our approach of using one year of midge arrival time data for calibration and a further two years data for validation of the midge dispersal sub-model has allowed an overall qualitative validation, but does not constitute a comprehensive quantitative validation.

The modelling methodology presented here adopted a simplified representation of *C.brevitarsis* habitat. Cattle free areas were denoted as being unable to support midge populations and thus any midge incursions into these areas failed to establish a resident population. This vector habitat representation was binary; each cell was either suitable for midge establishment due to the presence of cattle, or not. A more refined version would capture variation in cattle density and map this at greater spatial detail.

For practical applications of this spatially-explicit modelling methodology, more accurate wind models may be used. These provide gridded meteorological data down to 1 to 4 km resolution; examples include NAME (UK Met. Office [60]) and HYSPLIT_4 (Bureau of Meteorology, Australia). Both of these wind models have been used to model actual and possible wind dispersal of animal diseases; NAME used for BTV dispersal via *Culicoides* movement [13] and for Foot and Mouth Disease virus particle spread [61] and HYSPLIT_4 for potential BTV spread via *Culicoides* movement [15] and potential Foot and Mouth Disease virus plume spread [62].

## Conclusions

This study has introduced a spatially-explicit, discrete-event simulation modelling methodology to capture insect dispersal, via wind and flight, coupled with the establishment and growth of the population after arrival in areas with suitable habitat. The feasibility of this modelling technique has been demonstrated and shown to successfully capture the interrelated dynamics of insect population dispersal and establishment. Specifically, the methods developed have been demonstrated by their ability to replicate published spatio-temporal incursion data of *C.brevitarsis* obtained from extensive trapping programs. The modelling system is heavily parameterised, making it applicable to a range of pathogen carrying insect species by altering parameters appropriately.

The model described in this paper is intended to form the basis for an extended model that simulates the spread of Bluetongue virus, which will require the addition of host-to-vector and vector-to-host pathogen transmission sub-models. This extended model may then be used to address the effectiveness of intervention measures for mitigating BTV spread, such as spatially targeted vaccination strategies or movement bans or the impact of climate change on the risk and extent of disease outbreaks.

## Supporting Information

Text S1 **Additional model details and sensitivity analysis results.**
(DOCX)

Video S1 **Animated map of the population density during a low-temperature survival calibration simulation.**
(MP4)

Video S2 **Animation of the simulated population dynamics and midge spread for the 1991–1992 summer season.**
(MP4)
