# Supplementary material for: A Spatial Simulation Model for the Dispersal of the Bluetongue Vector Culicoides brevitarsis in Australia
Source: PLoS One. 2014 Aug 8;9(8):e104646. doi: 10.1371/journal.pone.0104646 (PMC4126746; doi:10.1371/journal.pone.0104646)
Supplement: Text S1 — Additional model details and sensitivity analysis results. (DOCX) [file pone.0104646.s001.docx]

**A Spatial Simulation Model for the Dispersal of the Bluetongue Vector *Culicoides brevitarsis* in Australia**

**Supporting Information Text S1**

**Table S1.1** : Landscape cell physical characteristics

| **Data** | **Type and Units** | **Description** |
| --- | --- | --- |
| *geographic data* | | |
| latitude | reals, decimal degrees | Location of nominal centre of cell |
| longitude |  |  |
| altitude | real, meters | height above sea level |
| area | real, square meters | cell area |
| *weather data* | | |
| temperature | time series: degrees Celsius | mean daily temperature |
| wind | time series: vector of triples: speed (km/h), direction (° clockwise form N) and duration (hours) | a set of wind speed, direction and duration triples, characterizing the main wind behaviour for each 3-hour period |

**Table S1.2** : Landscape cell biological characteristics

| **Data** | **Type and Units** | **Description** |
| --- | --- | --- |
| *vector data* | | |
| vector habitat | boolean | true if cell conditions allow a vector population |
| adult *Culicoides* density | real | relative population density, scaled so that 100 = effective maximum density of immature vectors |
| immature *Culicoides* density |  |  |

**Simulation Algorithm Outline**

1. Load adult and immature population data into each landscape cell from adult and immature population density map.
2. For each simulation day
   1. For each cell C
      1. Update cell mean daily temperature from weather data set
      2. Update insect density using population model
         1. Calculate numbers of: immature added through oviposition, adults added through maturation, immature and adults removed through death
         2. Update adult and immature population density using changes calculated in previous step
   2. Update adult density using diffusive movement model
      1. Make a temporary map M of cell tracking changes due to diffusive movement, initialise each cell in M to zero
      2. For each (unordered) pair of adjacent cells C1 and C1, calculate the net transport of adults between C1 and C1 and accumulate the changes in M
      3. For each cell C, update midge numbers using accumulated midges arriving from other cells as recorded by M
   3. For each 3-hour period within a 24 hour period
      1. For each cell C update wind speed and wind direction
      2. For each cell C, calculate number of midges becoming airborne, move midges from ground to airborne state
      3. Make a temporary map M of cells tracking changes in airborne adult density, initialise each cell in M to zero
      4. For each cell C
         1. Calculated downwind footprint from C based on wind speed and direction.
         2. Move airborne midges from C into downwind footprint cells into temporary map M, conserving midge numbers
      5. For each cell C, update airborne midge numbers using accumulated midges arriving from other cells as recorded by M
      6. For each cell C, calculate number of landing midges, move midges from airborne to grounded state.
   4. Output a snapshot of the population density of adults and immature of all cells

**Population Dynamics Model Summary**

**Table S1.3: Symbols used in population dynamics sub-model description**

| *symbol* | | *description* |
| --- | --- | --- |
| *variables* | | |
| *p_i_ , p_a_* | population, immature and mature (adult) | |
| *t* | time | |
| *parameters* | | |
| *b* | birth (oviposition) rate, function of temperature | |
| *d_i_ , d_a_* | death rate, function of temperature | |
| *m* | maturation (emergence) rate, function of temperature | |
| ε_i_, ε_a_ | temperature at below which declining populations become extinct, immature and adult | |
| *p_imax_* | immature population density at which oviposition becomes zero | |

The rate parameters *b*, *d* and *m* are assumed to be functions of temperature. Simple piecewise linear functions are used, as described in Table SI4.

**Table S1.4: *C. brevitsarsis* biological parameters**

| *parameter* | *temperature range* | *parameter value or range* | *source* |
| --- | --- | --- | --- |
| *b* | *below 7°C | 0 | [1-3] |
|  | *7°C to 18°C | 0 – 1.1 eggs/day |  |
|  | 18°C to 25°C | 1.1 – 3.96 eggs/day |  |
|  | above 25°C | 3.96 eggs/day |  |
|  |  |  |  |
| *d_i_* | *below 7°C | 1.0 (1 day) | [1] |
|  | *7°C to 17°C | 0.0328 (30.5 days) |  |
|  | above 17°C | 0.0328 (30.5 days) |  |
| *d_a_* | below 12°C | 0.1 (10 days) | [3] |
|  | 12°C to5°C | 0.45 (2.2 days) |  |
|  |  |  |  |
| *m* | *below 7°C | 0 | [1] |
|  | *7°C to 18°C | 0.027 (37 days) to 0.091 (11 days) |  |
|  | above 18°C | 0.091 (11 days) |  |
|  |  |  |  |
| ε_i_ |  | 0.001 |  |
| ε_a_ |  | 0.0005 |  |

* The value of 7°C appearing hear is the value of the low temperature survival parameter (LTAP) – the calibration of this value based on C.brevitarsis climatic zones is described in the main manuscript.

**Additional Information on Diffusive Movement Model**

We implemented the forward Euler numerical solution to the diffusion equation, calculating the net change in population density in each cell using diffusion coefficient and the population density gradient between each cell and its four nearest neighbours. Simple von Neumann stability analysis indicated that the chosen spatio-temporal resolution (~5 km cell spacing, and 24-hour time steps) and diffusion coefficient should be numerically stable, and this was verified by simulation experiment.

The use of a 4-cell (von Neumann) neighbourhood instead of an 8-cell (Moore) neighbourhood does introduce short-range, temporary artifacts in spatial population density, since it takes two simulation cycles for diffusing midges to reach corner-adjacent cells. However over a time span of 2 or more days, these artifacts are smoothed out and diffusing midge densities in each cell rapidly approach the correct values as defined by a continuous-space and –time diffusion process. This can be seen in Figures S1.1 below, where a smooth circular diffusion pattern is exhibited despite the discreteness and 4-cell neighbourhood of the underlying grid. Since the other processes that depend upon the population density (i.e. population dynamics processes) occur with characteristic timescales of multiple days, the temporary artifacts introduced by diffusion do not impact on simulation outcomes.

**Sub-Model Calibration and Sensitivity Analyses Results**

For the results reported in this section, agreement between simulated and observed midge arrival times is measured by Cohen’s kappa statistic, with disagreements between weighted by the square of the number of months difference between observed and simulated arrival. Cohen’s kappa ranges from 1.0 indicating perfect agreement to 0.0 indicating a level of agreement expected by chance alone; with negative values indicating systematic disagreement. The significance value p is the probability of observing the level of agreement (kappa value) assuming that the true agreement is zero i.e. that simulation results are essentially random. Unless otherwise stated, observations and simulations are for the NSW trapping sites 1991/2, the model parameter values are as given in the main paper, and the process used to quantitatively compare simulated and observed spreads is as described in Section 2.4 “Quantifying agreement between simulated and observed midge spread”.

**Table S1.5 : Wind transport sub-model calibration**

|  | **trapping detection threshold** | | | | | |
| --- | --- | --- | --- | --- | --- | --- |
|  | **0.005** | | **0.05** | | **0.5** | |
|  | **kappa** | **p** | **kappa** | **p** | **kappa** | **p** |
| **wind factor** |  |  |  |  |  |  |
| x1 | 0.117 | 0.0662 | 0.103 | 0.0891 | 0.0908 | 0.074 |
| x2 | 0.118 | 0.0571 | 0.118 | 0.0571 | 0.161 | 0.0305 |
| x3 | 0.0539 | 0.573 | 0.519 | 0.00202 | 0.332 | 0.0115 |
| x4 | 0.37 | 0.00618 | 0.527 | 0.00485 | 0.547 | 0.00639 |
| x5 | -0.155 | 0.0328 | 0.408 | 0.0233 | 0.443 | 0.0264 |

Table S1.5 gives the agreement between simulated and observed midge arrival times for five different values for the parameter used to calibrate the maximum wind transport speed parameter (described in the main paper in sections 2.3 “Wind-borne dispersal” and 2.4 “Wind transport sub-model calibration). The parameter value is a multiplier for the automatic weather station wind speed (recorded at 10m above ground) that gives the maximum speed at which midges are dispersed by the wind. A multiplier of 4.0 (x4) gave the best match to the observed midge spread. We repeated the parameter calibration for two alternative values of the trapping detection threshold parameter, which is the value above which the simulated population must rise in order to be first detected in a landscape cell. As can be seen from the row shaded in grey, the x4 multiplier value was the best fit over two orders of magnitude of the trapping threshold parameter value, indicating that the calibrated value is robust to the value of the trapping threshold parameter.

**Table S1.6 : Lofting rate sensitivity analysis**

| **lofting rate (day^-1^)** | **agreement (kappa)** | **significance (p)** |
| --- | --- | --- |
| 0.1 | 0.583 | 0.00378 |
| 0.5 | 0.512 | 0.00818 |
| 1.0 | 0.527 | 0.00485 |
| 2.0 | 0.333 | 0.0843 |
| 10.0 | 0.353 | 0.0692 |

Table S1.6 shows the agreement between observed and simulated midge arrival times at for alternative values of the lofting rate parameter ranging from 0.1 to 10 (the value assumed in the model was 1.0). It can be seen that values smaller than the model assumption make little difference to the agreement, but that larger values result in less accurate simulations (with the overall rate of midge spread being overestimated).

**Table S1.7 : Landing rate sensitivity analysis**

| **landing rate (day^-1^)** | **agreement (kappa)** | **significance (p)** |
| --- | --- | --- |
| 0.1 | 0.527 | 0.00485 |
| 0.5 | 0.527 | 0.00485 |
| 1.0 | 0.527 | 0.00485 |
| 2.0 | 0.495 | 0.00933 |
| 10.0 | 0.595 | 0.00325 |

Table S1.7 shows the agreement between observed and simulated midge arrival times at for alternative values of the land rate parameter ranging from 0.1 to 10 (the value assumed in the model was 1.0). It can be seen that the model is relatively insensitive the landing rate parameter.

**Table S1.8 : No-dispersal (overwintering model)**

|  | **trapping detection threshold** | | | | | |
| --- | --- | --- | --- | --- | --- | --- |
|  | **0.005** | | **0.05** | | **0.5** | |
|  | **kappa** | **p** | **kappa** | **p** | **kappa** | **p** |
| **LTAP*** |  |  |  |  |  |  |
| -5°C | -0.0725 | 0.179 | -0.404 | 0.0449 | 0.317 | 0.0441 |
| 0°C | -0.126 | 0.272 | -0.364 | 0.0565 | 0.153 | 0.384 |
| 5°C | 0 | - | 0 | - | 0 | - |

*low temperature activity parameter – see main manuscript Section 2.2 for definition

Table S1.8 shows the results simulations of an alternative hypothesis (described in the main paper in Section 3.4) whereby the apparent “arrival times” of midges along the NSW coast is actually due to overwintering populations being present in all areas. For these simulations, the midge dispersal sub-models were turned off. All landscape cells were seeded with adult and immature populations at the beginning of 1991, and the population dynamics of each cell was allowed to evolve according to local daily temperature until 30^th^ September 1991. The standard assessment of simulated with observed spread was then conducted. Three alternative values for the LTAP were examined (all being lower than the main model LTAP, allowing overwintering in more southerly locations), along with two alternative detection threshold parameters values (see notes on Table S1.5 above).

Consistent with what is understood from studies of *C.brevitarsis* populations in NSW [4-8], we found that this “no-dispersal” model was not capable of plausibly reproducing the observed pattern of midge detection. The only model that produced a statistically significant, positive correlation between observed and simulated “arrival” times requires some degree of oviposition, maturation, and immature survival below 0°C (i.e. LTAP of minus 5°C), which is not physically plausible [1].

**Table S1.9 : Diffusion-only model**

| **diffusion**  **coefficient**  **(m^2^/s)** | **comment** | **agreement (kappa)** | **significance (p)** |
| --- | --- | --- | --- |
| 7.5 | Half *C.variipennis* value | -0.001 | 0.904 |
| 12.96 | *C.variipennis* value (used in main model) | -0.005 | 0.711 |
| 60.11 | *C.impunctatus* value | 0.0168 | 0.672 |
| 120 | twice *C.umpuncatus* value | 0.088 | 0.226 |

Table S1.8 shows the agreement between observed and simulated midge arrival times for alternative models that do not include the wind-borne part of the midge transport sub-model. As can be seen, none of the diffusion-only models approach the level of agreement of the full model, even with a diffusion coefficient twice as large as the highest value found for *Culicoides* species found in the literature.

**Table S1.10 : Constant temperature model**

| **temperature (°C)** | **agreement (kappa)** | **significance (p)** |
| --- | --- | --- |
| 15 | 0 | - |
| 18 | 0.525 | 0.00431 |
| 20 | 0.462 | 0.00848 |
| 25 | -0.243 | 0.0175 |

Table S1.10 shows the agreement between observed and simulated midge arrival times for an alternative model where temperature was constant and spatially uniform. A constant temperature model (using a temperature of 18°C) performs as well as the full model in terms of predicting the seasonal midge arrival times. However as discussed in the main paper in Section 3.4, constant temperature models no not predict the declining populations in the autumn and winter months, and thus do not leave the population density map in a realistic state at the end of a seasonal simulation, precluding their use for multi-year simulations.

**Combined Population and Dispersal Dynamics**

B

A


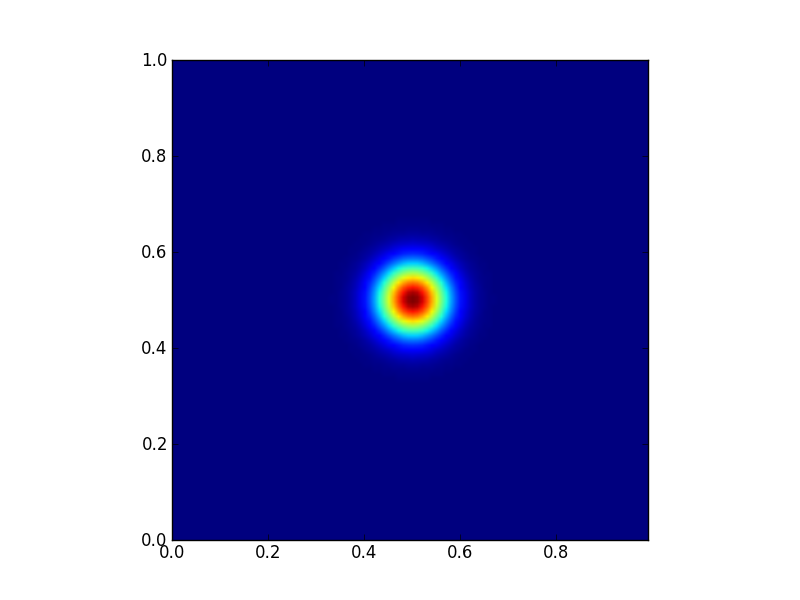

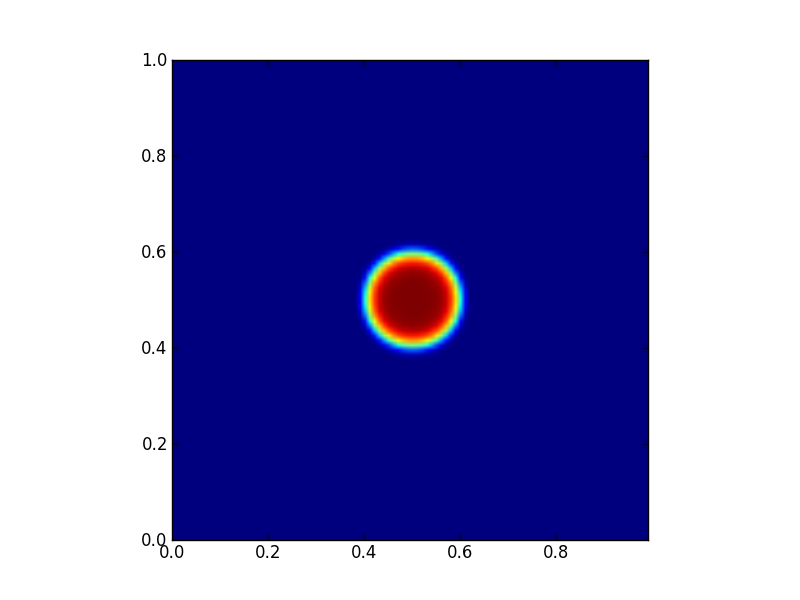

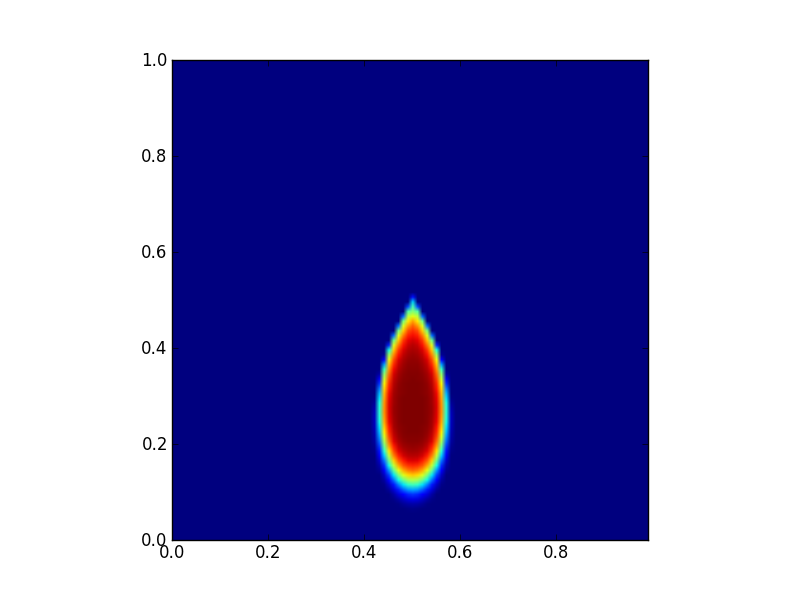


C

**Figure S1.1** : **Combined population dynamics, diffusion and dispersal.**

A: Diffusive spread, no population growth

B: Diffusive spread with population growth

C: diffusive spread, population growth and (northerly) wind dispersal.

Figure S1.1 shows the operation of the population dynamics and transport sub-models in an idealised environment. A 100x100 cell grid is used, and model parameter values have been selected to demonstrate the following phenomena. Three scenarios are pictured. In (A), an initial single cell population diffuses over time, but without any population growth dynamics: the population is constant and just spreads over a greater area. With (B) the same diffusion process operates, but population dynamics are also in effect. Notice that the central population is maintained at a high level, and that the population has diffused further than the top-left scenario, since the higher central population maintains a higher rate of diffusion. On bottom panel (C), the same point population undergoes population dynamics and also wind-driven dispersal by a constant northerly wind. The result is similar to combined diffusion and population dynamics, but the directional nature of wind dispersal has created a teardrop-shape oriented downwind.

**Figure S1.2 : Location of study area and automatic weather stations**

The area used in this study is shown in the blue box; Australian Bureau of Meteorology automatic weather station locations area shown as blue dots (all stations in the study areas were used as a source of wind data).

**References**

1. Bishop AL, McKenzie HJ, Barchia MI, Harris AM (1996) Effect of Temperature Regimes on the Development, Survival and Emergence of Culicoides brevitarsis Kieffer (Diptera: Ceratopogonidae) in Bovine Dung. Australian Journal of Entomology 35 361-368.

2. Campbell MM (1975) Oogenesis in Culicoides brevitarsis Kieffer (Diptera: Ceratopogonidae) and the Development of a Plastron-like Layer on the Egg. Australian Journal of Zoology 23: 203-218.

3. Gerry AC, Mullens BA (2000) Seasonal Abundance and Survivorship of Culicoides sonorensis (Diptera: Ceratopogonidae) at a Southern California Dairy, with Reference to Potential Bluetongue Virus Transmission and Persistence. Entomological Society of America 37: 675 - 688.

4. Bishop AL, McKenzie HJ (1994) Overwintering of Culicoides spp. (Diptera: Ceratopogonidae) in the Hunter Valley, New South Wales. Journal of the Australian Entomological Society 33: 159-163.

5. Bishop AL, Barchia IM, Harris AM (1995) Last occurrence and survival during winter of the arbovirus vector Culicoides brevitarsis at the southern limits of its distribution. Australian Veterinary Journal 72: 53-55.

6. Bishop AL, Kirkland PD, McKenzie HJ, Spohr LJ, Barchia IM, et al. (1995) Distribution and Seasonal Movements of Culicoides brevitarsis Kieffer (Diptera: Ceratopogonidae) at the Southern Limits of its Distribution in New South Wales and their Correlation with Arboviruses Affecting Livestock. Journal of the Australian Entomological Society 34: 289-298

7. Murray MD (1991) The Seasonal Abundance of Female Biting-midges, Culicoides bvevitavsis Kieffer (Diptera : Ceratopogonidae), in Coastal South-eastern Australia. Australian Journal of Zoology 39: 333-342.

8. Murray MD, Nix HA (1987) Southern Limits of Distribution and Abundance of the Biting-Midge Culicoides brevitarsis Kieffer (Diptera : Ceratopogonidae) in South-Eastern Australia: an Application of the GROWEST Model. Australian Journal of Zoology 35,: 575-585.
